# Health policy implications of emerging infections.

**DOI:** 10.3201/eid0403.980309

**Published:** 1998

**Authors:** K Hein

**Affiliations:** Institute of Medicine, National Academy of Sciences, Washington, D.C., USA.


### Health Policy Implications of Emerging Infections

**Karen Hein**
Institute of Medicine, National Academy of Sciences, Washington, D.C., USA

The solutions to emerging disease problems involve politics and policy issues, as well as solid science. The National Academy of Sciences' Institute of Medicine (IOM), whose mission is to "improve the health of people of the nation and the world," draws upon the expertise of elected members as well as others in the United States and other nations to make policy recommendations. Groups convene to debate contentious issues and publish evidence-based reports with recommendations to government, academia, industry, and the public.

Evidence-based reports are the foundation upon which policy can be built. In this last decade, IOM has produced several documents that have focused on emerging infections and provided a springboard for policy on a local, nationwide, and international scale. The U.S. Capacity to Address Tropical Infectious Disease Problems (1987) (1) concluded that U.S. capacity was barely adequate and that improvement in policies and modest additional funding could make a substantially stronger contribution to the field. Required efforts included sustained support for basic and applied research; accelerated development and testing of new preventive, therapeutic, and diagnostic technologies; sustainable career structures for tropical disease professionals; increased capacity to train U.S. tropical disease professionals and those from developing countries in research and public health service; development of disease surveillance capabilities; strengthened institutional capabilities in developing countries; and flexible, responsive administration of programs.

The Future of Public Health (1988) (2) report made three basic recommendations regarding the mission of public health and defined its core functions to be assessment, policy development, and assurance. It also included guidance for the government's role in fulfilling the public health mission and the responsibilities unique to each level of government. The report has been a useful blueprint for the past decade.

Emerging Infections: Microbial Threats to Health in the United States (1992)(3) identified significant emerging infectious diseases, determined what might be done to deal with them, and recommended how similar future threats might be confronted to lessen their impact on public health. The document focused on factors contributing to disease emergence, not the diseases themselves: human demographics and behavior, technology and industry, economic development and land use, international travel and commerce, microbial adaptation and change, and the breakdown of public health measures.

Sexually Transmitted Diseases: The Hidden Epidemic (1997) (4) focused on the need for a new social norm of healthy sexual behavior. The small investment in prevention efforts was contrasted with the very high costs of care for treating sexually transmitted diseases (STDs) (Figure 1). The report also examined the obstacles and opportunities presented by managed care. Limitations include the low priority for STD prevention, emphasis on short-term cost savings,

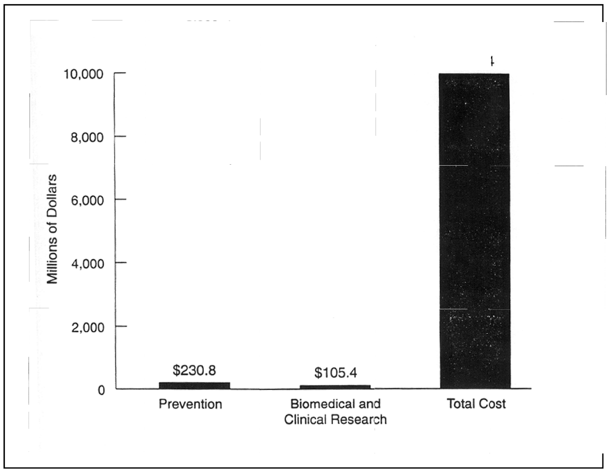

Figure 1. Estimated annual direct and indirect costs for selected sexually transmitted diseases (STDs) and their complications in 1994 versus national public investment in STD prevention and research in federal fiscal year 1995 (4).

varying technical capabilities for diagnosis and treatment, and patient concerns about confidentiality and treatment of partners not enrolled in the same health plan. Lastly, opportunities for training and continuing education in STD control and prevention are not built into most managed care settings. The report called for several steps including a national campaign to heighten awareness of the human and financial costs of STDs and to promote the use of social marketing techniques for their prevention. A recent innovative informational campaign used a niche approach and a social marketing strategy with the spot video Hittin' the Skins and the public service announcement Knockin' Boots (D. Futterman, pers. comm.), geared toward alerting 16- to 21-year-olds of the need for HIV testing.

Many related activities, in addition to the IOM reports, have underscored the danger of emerging infectious diseases and reiterated the warnings about the overall erosion of the U.S. public health system during the 1990s. The reports also provided specific, detailed recommendations for action by individual agencies. In 1994, the Centers for Disease Control and Prevention (CDC) published Addressing Emerging Infectious Disease Threats: A Prevention Strategy for the United States. In the same year, the U.S. National Science and Technology Council's Committee on International Science, Engineering, and Technology (an interagency working group) was convened to consider the global threat of emerging and reemerging infectious diseases and in 1995 published the report Infectious Disease—A Global Health Threat. In 1995, the National Security Council asked the federal government to examine its preparedness to respond to global epidemics.

In 1995, the Food Safety and Inspection Service, CDC, and the Food and Drug Administration (FDA) developed the Sentinel Site Study, which evolved into FoodNet and now includes collection of more precise information on the incidence of foodborne disease in the United States. In 1996, President Clinton's administration set out a new policy to establish a worldwide infectious disease surveillance and response system and expand certain federal agency mandates to better protect American citizens.

In the 1996 NIAID Research Agenda for Emerging Infectious Diseases, the National Institutes of Health described research and training issues relevant to the national strategy for confronting the threat of emerging and reemerging infections and related its approach to addressing these issues.

In 1996, the Department of State established an Emerging Infectious Diseases and HIV/AIDS Program to serve as a focal point for the development and implementation of U.S. foreign policy objectives to improve the health of U.S. citizens and to stem the spread of infectious diseases worldwide through various international bilateral and multilateral negotiations. This program has received $50 million in funding. Other government agencies, including FDA, U.S. Agency for International Development, Department of Defense, National Oceanic and Atmospheric Administration, National Aeronautics and Space Administration, and U.S. Department of Agriculture, have also examined the issue of U.S. vulnerability to epidemics and resurgence of infectious disease threats.

The IOM's Forum on Emerging Infections is the most recent activity within the National Academy of Sciences to keep sustained attention on these issues. The forum was established in 1996 to provide a structured opportunity for discussion and to scrutinize critical, and possibly contentious, scientific and policy issues related to research on and the prevention, detection, and management of new and reemerging infections. The forum has organized a series of workshops to be conducted over 30 months. Workshop topics include costs of infectious diseases, surveillance, antimicrobial resistance, effects of health-care restructuring on public health and basic research related to infectious diseases, capacity for emergency response to emerging and reemerging infectious diseases, education and training needs, predicting the future, and behavioral interventions.

Orphans and Incentives (5), a 1998 report, is the first publication of the forum; it focused on constraints that have left an undefined group of "urgently needed medical products in an orphaned condition which demands special attention." The authors examined these products across the product cycle and then classified them into categories for which incentives might be developed to bolster the competitiveness of such products in industrial portfolios.

The 1998 report Antimicrobial Resistance (6), the second publication of the forum, examined increases in the number of pathogens, multidrugresistant strains, compromised persons (including HIV-infected patients), deaths from infection with resistant organisms, speed of the global spread, and costs of health care. The report also examined decreases in the antimicrobial armamentarium, amount of research and development expended when resistance was not seen as a major threat, and funding for public health infrastructure and addressed the following topics: expansion, coordination, and improvement of the diverse elements of surveillance; need for relatively small but thoughtful investments in research, clinical management and practice, and policy; use of antibiotics in food production; ways to prolong the effectiveness of existing antibiotics; basic research and incentives for new antibiotics; and legal and regulatory mechanisms in key areas of need.

A soon-to-be-published report on a March 1998 workshop on managed care will examine the implications of managed care systems on emerging infections by reviewing basic and clinical research, clinical practice guidelines, surveillance and monitoring, prevention, education and outreach, and product development.

These reports and events have examined research on emerging infectious diseases and crafted a series of policy recommendations. They put forth a rationale for why the United States should invest in global health. The 1997 report, entitled America's Vital Interest in Global Health (7), provided a new framework for thinking about the benefits to the United States, as well as to the rest of the world, of our increased participation. The movement of two million people each day across national borders and the growth of international commerce are inevitably associated with transfers of health risks (e.g., infectious diseases, contaminated food, terrorism, and legal or banned toxic substances). U.S. commitment to global health serves to protect our people, enhance our economy, and advance our international interests. Moreover, governments are no longer the sole agents in the global health arena (Figure 2).

The United States can contribute not only with funding, but also with the scientific and technical expertise in its health sector. The United States should lead from its strengths (medical science and technology) in the areas of research and development, surveillance, education and training, global partnerships, and coordination and leadership. In this way, the United States "can do well by doing good."

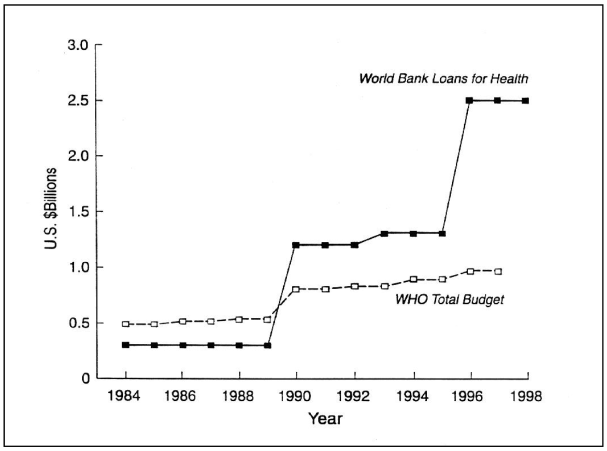

Figure 2. The growing role of the World Bank in health (7).

### Acknowledgments

The author thanks Carol Bock for her assistance in preparing this manuscript and Jonathan Davis for collecting background materials.

### References

1. The U.S. capacity to address tropical infectious disease problems. Washington: National Academy Press; 1987. p. 88. Sponsored by the Board on Science and Technology for International Development, Office of International Affairs, National Research Council and Institute of Medicine, National Academy of Sciences.
2. The future of public health. Washington: National Academy Press; October 1988. p. 240. Sponsored by the Institute of Medicine, Division of Health Care Services.
3. Lederberg J, Shope RE, Oaks SC Jr, editors. Emerging infections: microbial threats to health in the United States. Washington: National Academy Press; October 1992. p. 312. Sponsored by the Institute of Medicine, Division of Health Sciences Policy and Division of International Health.
4. Eng TR, Butler WT, editors. The hidden epidemic: confronting sexually transmitted diseases. Washington: National Academy Press; 1997. p. 392. Sponsored by the Institute of Medicine, Board on Health Promotion and Disease Prevention.
5. Harrison PF, Lederberg J, editors. Orphans and incentives: developing technology to address emerging infections, Workshop Report. Washington: National Academy Press; 1997. Sponsored by the Institute of Medicine.
6. Antimicrobial resistance: issues and options. Workshop Report. Washington: National Academy Press; 1998. Sponsored by the Institute of Medicine, Forum on Emerging Infections.
7. America's vital interest in global health, protecting our people, enhancing our economy, and advancing our international interests. Washington: National Academy Press; 1997. Sponsored by the Institute of Medicine, Board on International Health.

